# *Tripterygium wilfordii* ameliorates complete freund adjuvant-induced rheumatoid arthritis through modulating the gut-joint axis

**DOI:** 10.3389/fimmu.2026.1828110

**Published:** 2026-10-08

**Authors:** Caili Zhang, Qingru Chen, Zheng Sun, Tiantian Wu, Yujie Zhang, Binbin Huang, Shenghai Huang, Yan Zhang, Maozhen Han

**Affiliations:** 1 School of Life Sciences, Anhui Medical University, Hefei, Anhui, China; 2 Microbial medicinal resources development research team, Anhui Provincial Institute of Translational Medicine, Hefei, Anhui, China; 3 School of Public Health, Anhui Medical University, Hefei, Anhui, China; 4 Department of Microbiology, The Key Laboratory of Microbiology and Parasitology of Anhui Province, The Key Laboratory of Zoonoses of High Institutions in Anhui, School of Basic Medical Sciences, Anhui Medical University, Hefei, Anhui, China; 5 School of Life Sciences, Hefei Normal University, Hefei, Anhui, China

**Keywords:** gut microbiota, multi-omics, network pharmacology, rheumatoid arthritis, Th17/Treg balance

## Abstract

**Introduction:**

Although *Tripterygium wilfordii* (TW) is a classical Chinese herbal medicine for the treatment of rheumatoid arthritis (RA), the mechanism by which it alleviates inflammation through modulation of the gut microbiota remains unclear.

**Methods:**

To elucidate the mechanism underlying the therapeutic effects of TW on RA, we first employed network pharmacology to predict the relevant pathways and core targets. Subsequently, an integrative multi-omics approach was applied, including gut metagenomics, gut metabolomics, serum metabolomics, and synovial transcriptomics, to investigate the alterations in gut microbiota, intestinal and serum metabolites, and synovial gene expression in a rat model of RA.

**Results:**

Our findings revealed that TW supplementation lowered serum concentrations of IL-6, IL-1β, TNF-α, and IL-10 and modulated the Th17/Treg balance in intestinal tissue. TW treatment altered gut microbiota (GM) composition, marked by increased abundance of *Prevotella* sp *PCJ2* and *Psychrobacter pasteurii*, and decreased *Adlercreutzia equolifaciens* and *Schaedlerella arabinosiphila*, synergizing with keystone species (*Bifidobacterium animalis, Phocaeicola sartorii, Blautia faecis*) to promote gut health. Concurrently, TW elevated tryptophan metabolites (tryptophol, tryptamine, 5-hydroxykynurenamine) while reducing 4-(2-aminophenyl)-2,4-dioxobutanoic acid, restoring tryptophan metabolism. This regulation coincided with upregulated *Inmt* mRNA in synovium, activating the tryptophan pathway. Correlation and mediation analyses indicated that gut-derived circulating metabolites such as N-myristoyl arginine and lauramidoxide were positively correlated with synovial *Jun* expression, potentially participating in the modulation of the IL-17-NF-κB cascade. Consistently, upregulated synovial *Socs3* may exert negative feedback suppression on JAK-STAT signaling to alleviate inflammatory responses.

**Discussion:**

These findings point to the gut microbiome and tryptophan pathway as a new therapeutic avenue for RA.

## Graphical Abstract

**Figure:**
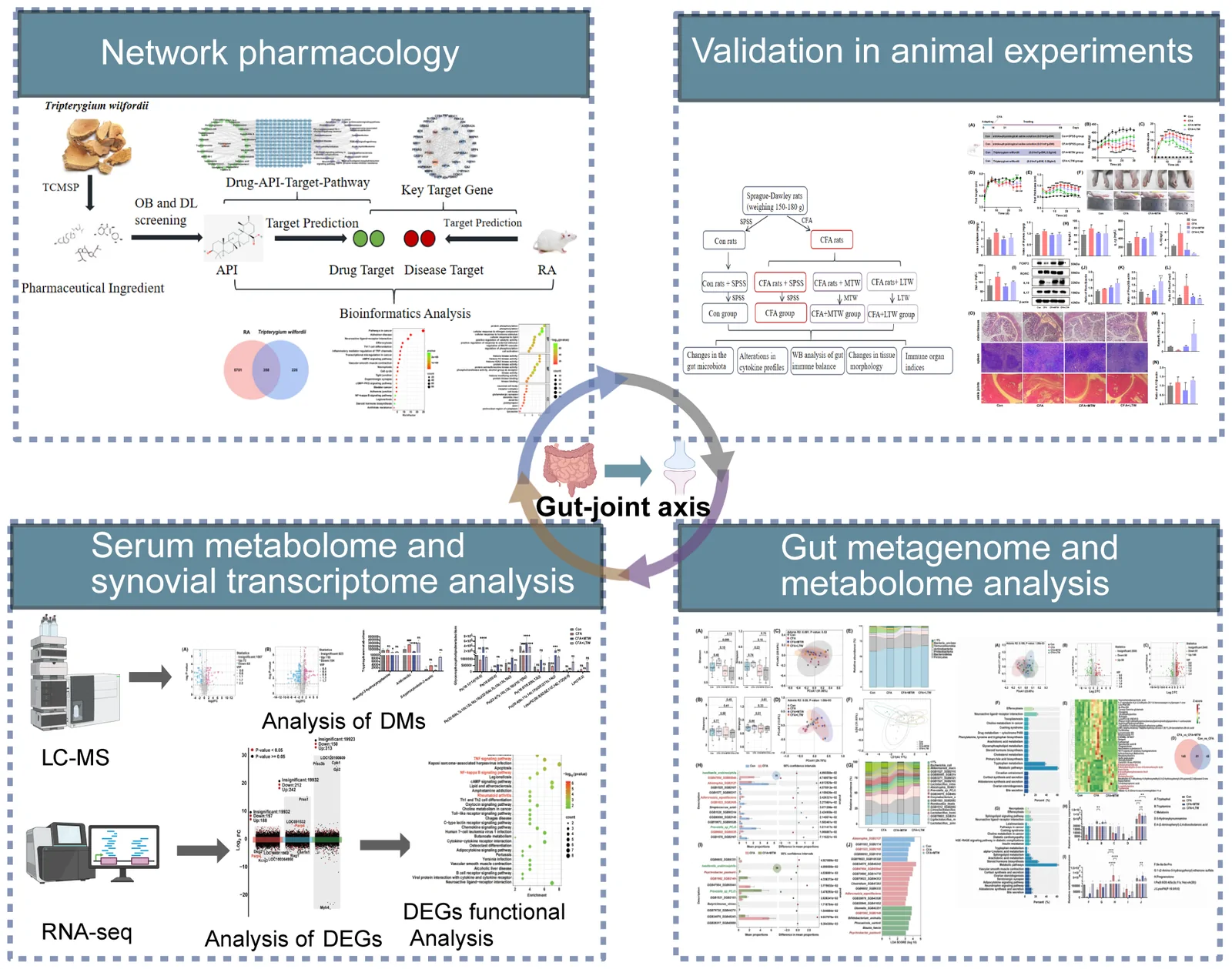

## 1 Introduction

RA is a chronic inflammatory and autoimmune disease marked by debilitating polyarticular inflammation associated with bone and cartilage erosions, which culminates in joint deformity and functional deficits (1). RA affects approximately 1% of the adult population globally (2). It is currently considered to result from the multifactorial interplay of various factors, including hereditary factors, bacterial infections, neuroendocrine factors, and immune system abnormalities. While the pathogenesis of RA is not entirely clear, studies indicate that T cell immune dysregulation and gut microbiota disorder are crucial factors in its development. Therefore, it is essential to elucidate the gut-joint interactions mediated by the digestive and immune systems. It can facilitate the discovery of new therapeutic strategies for RA and promote translational applications in gut microecology research.

Accumulating evidence indicates that the gut microbiota and its metabolites play a pivotal role in the pathogenesis of RA (3–5). Gut microbiota disorder can cause gut barrier dysfunction (6), thereby triggering dysregulation of both innate and adaptive immune responses in the host. Consequently, GM modulation has become a viable strategy for RA therapy. For example, several specific microbial species, such as *Prevotella copri* and *Lactobacillus casei* (7), *Faecalibacterium prausnitzii* (8), *Lactobacillus plantarum* (9), *Bifidobacterium breve* (10), and *Bacteroides fragilis* (11) can effectively alleviate synovitis, joint swelling, and joint erosion. GM-derived metabolites, including short-chain fatty acids, tryptophan metabolites, bile acids, and amino acid metabolites, are pivotal regulators of immune responses. They operate by adjusting immune cell activity and inhibiting the production of pro-inflammatory cytokines. Therefore, restoring microbial equilibrium to optimize the production of these metabolites represents a promising therapeutic avenue for RA (12, 13).

Strategies for reshaping the gut microbiome extend beyond probiotics (14) to encompass traditional Chinese medicines, like TW, known to influence microbial structure and activity. The main bioactive components of TW are diterpenoids and alkaloids. TW exhibits multiple pharmacological effects, primarily including anti-inflammation, analgesia, and immune regulation. Growing evidence indicates that the effect of TW on RA is mainly achieved by regulating immune cells, inflammatory cytokines, and inflammation-related signaling pathways (15–17). Additionally, it can regulate the composition of the gut microbiota to improve inflammation-related diseases (18). Therefore, the beneficial effects of TW on RA may be partially attributed to the role of the gut microbiota. Nonetheless, additional research is needed to identify the distinct gut microbial and pivotal microbial byproducts linked to TW’s anti-inflammatory impact.

Thus, this research sought to clarify how TW alleviates RA through gut microbiota interactions. We conducted a comprehensive analysis using network pharmacology, animal experiments, and multi-omics approaches. We focused on gut microbiota, their metabolites, and the impacts of these metabolites on joint synovial gene expression. In this study, oral administration of TW to Complete Freund Adjuvant (CFA)-induced RA rats were used to treat RA. Effects were evaluated by comparing with normal and positive control groups. During the intervention, body weight, paw length and thickness, and arthritis indices of rats were monitored to directly assess TW’s therapeutic effects. Fecal samples were gathered pre-modeling (week 2), after successful modeling (week 4), and post-intervention (week 9) for comprehensive liquid chromatography-mass spectrometry (LC-MS) and metagenomic sequencing to evaluate alterations in gut metabolites and gut microbial communities. Serum metabolic profiles were evaluated via LC-MS analysis of collected blood samples. Spleen and thymus samples were collected, and ankle synovial tissue was used for transcriptome sequencing.

## 2 Materials and methods

### 2.1 Network pharmacology analysis to screen active ingredients of TW and target genes of RA

The chemical constituents of TW were retrieved from the traditional Chinese medicine systems pharmacology database and analysis platform (TCMSP). Screening was conducted based on the thresholds of oral bioavailability (OB) ≥ 30% and drug-likeness (DL) ≥ 0.18 (19). Subsequently, potential therapeutic targets of the active components from *Tripterygium wilfordii* were identified through the public chemical database (Pubchem). Three databases were used to search for therapeutic targets for RA: The GeneCards Database (https://previous.genecards.org), Online Mendelian Inheritance in Man (OMIM, https://omim.org) database, and Therapeutic Target Database (TTD, https://db.idrblab.net/ttd/).

### 2.2 Preparation of TW

For each 10 g of TW, 10 volumes of ultrapure water were added, and the TW was soaked for 2 hours. The soaked herb was then decocted twice: first for 30 minutes, followed by a second decoction for 40 minutes. The decoction liquids from both boils were combined and mixed thoroughly. The mixture was maintained at ambient temperature for 30 min, then filtered and concentrated to yield a final concentration of 0.5g crude drug per ml. The concentrated extract was refrigerated at 4 °C for later applications (20).

### 2.3 Experimental design of animals

A cohort of forty male, six-week-old Sprague-Dawley rats (weighing 150–180 g), certified as specific-pathogen-free (SPF), was obtained from Liaoning Changsheng Biotechnology Co. Ltd. The rats were maintained in a sterile environment at 22 ± 1 °C and 45–55% relative humidity. After a 2-week acclimatization period, rats were randomly allocated to either the control (Con, n=10) and CFA groups (n=30). Rats in the Con and CFA groups received tail vein injections of 0.1 mL saline and CFA emulsion (day 0) (21), respectively. Arthritis index severity was assessed every 2–3 days following the initial immunization. Using index values, body weight, and paw swelling metrics, CFA rats were categorized into CFA, CFA+MTW, and CFA+LTW groups on day 17. The CFA+MTW and CFA+LTW groups received TW traditional Chinese medicine preparation via oral gavage at doses of 5g/kg/d and 2.5g/kg/d, respectively. The Con and CFA groups received an identical dose (10 mL/kg) of stroke-physiological saline solution (SPSS) by gavage. These interventions were applied daily throughout the 28-day period. Upon completion of the study (day 45), fresh fecal samples were collected for microbial and metabolomic profiling. Urethane (20 g) was accurately weighed and added to 80 mL of sterilized distilled water. The mixture was shaken thoroughly and allowed to stand for 15 minutes to prepare the urethane anesthetic solution. Rats were kept in a temperature-controlled environment at 22–25 °C and given an intraperitoneal injection of urethane at a dose of 7.5 mL/kg. After the rats were adequately anesthetized, abdominal aortic blood was collected, and the spleen, thymus, joints, synovium, and colon were rapidly excised. Samples of the spleen and thymus were weighed immediately, snap-frozen in liquid nitrogen, and then stored at −80 °C until further analysis.

### 2.4 Assessment of rat arthritis index (AI) scores, body weight, paw length, and paw thickness

Throughout the modeling phase and the entire experimental period, body weight, paw dimensions, and AI scores assessments were recorded every three days. Concurrently, general conditions including diet, fur condition, feces, mental state, and spontaneous activity were observed. The AI score was assessed according to the following criteria: 0 = no changes in the interphalangeal joints and ankle joint. 1 = mild redness and swelling in parts of the toes and paw. 2 = partial toe and paw redness with swelling. 3 = severe redness and edema below the ankle. 4 = generalized severe joint swelling distal to the ankle. The AI scores for each rat was defined as the cumulative score from all four paws, yielding a maximum possible score of 16 (22).

### 2.5 Immunological organ indices, cytokine assays, and tissue pathology assessments

Following the intervention period, rats were anesthetized and euthanized. Spleen and thymus were weighed for each group. The immune organ index was calculated using the formula: Spleen or thymus (mg)/rat body weight (g). Serum levels of IL-17, IL-10, IL-6, IL-4, TNF-α, and IFN-γ were quantified using commercial ELISA kits (Reed Biotech Co., Ltd., Wuhan, China). Samples from the joint, spleen, and colon were collected for histopathological examination. Following fixation in 4% paraformaldehyde, the tissues were embedded in paraffin, and 5 μm sections were cut and stained with hematoxylin and eosin (H&E) according to a standard protocol (23).

### 2.6 DNA of fecal samples extraction, sequencing, and metagenome analysis

Fecal genomic DNA was extracted with the PowerSoil Kit, its purity analyzed via 1% agarose gel, and its quantity determined by a Qubit 3.0 Fluorometer. Paired-end libraries with a 350 bp insert were generated using the VAHTS Universal Plus DNA Library Prep Kit and underwent 150 bp sequencing on the BGI T7. The raw sequencing data underwent a standardized bioinformatics processing pipeline. Firstly, quality control and adapter trimming were carried out using fastp (v0.23.2) with default parameters. Secondly, for the removal of leftover host-derived sequences, the filtered sequences were mapped against the reference genome via Bowtie2 (v2.4.5). Thirdly, taxonomic profiling was performed with MetaPhlAn4 (v4.0.6) and the default marker gene database was utilized (24).

### 2.7 LC-MS analysis for fecal and serum samples

For metabolite extraction, 50 ± 5 mg of fecal sample was homogenized in a 2 mL tube with a grinding bead and 400 µL of methanol/water (4:1, v/v) solution containing internal standards. The samples were ground at -10°C for 6 min, extracted by cryogenic ultrasound (5°C, 40 kHz) for 30 min, and then centrifuged at -20°C (13,000 g, 4°C) for 15 min. Serum samples were extracted with 400 μL of a methanol: acetonitrile (1:1, v/v) solution. The samples were then subjected to 30 minutes of ultrasonic treatment at 5 °C at a 40 kHz rate. Following protein precipitation, the supernatants were dried down prior to loading. Supernatants underwent LC-MS analysis on a UHPLC-QExactiveHF-X system with an ACQUITY UPLC HSS T3 column. Elution was accomplished with specified solvent gradients: Phase A (95% water, 5% acetonitrile, 0.1% formic acid) and Phase B (47.5% acetonitrile, 47.5% isopropanol, 5% water, 0.1% formic acid). Essential MS settings included a 3.5 kV spray voltage and a 325 °C capillary temperature. Subsequent data processing for metabolite identification was carried out in Progenesis QI v3.0, requiring a mass accuracy of <10 ppm and confirmed by MS/MS matching (25).

### 2.8 Transcriptome analysis for joint synovial tissue

Total RNA was isolated from rat knee joint synovium using TRIzol reagent (Invitrogen). The enriched mRNA was then fragmented into approximately 200 bp segments, followed by cDNA synthesis with random primers. The ligated sequences underwent PCR amplification and purification for constructing the final library. The library was constructed using Illumina (HiSeq X Reagent kit, Illumina, San Diego, USA), and the library was sequenced using DNBSEQ-T7RS Reagent Kit. Adapter removal and quality filtering of raw reads were performed using fastp (v0.23.2). HISAT2 (v2.2.1) aligned the clean reads with the rat reference genome. Subsequently, the subread software (v2.0.2) was employed to accurately count the reads. Ultimately, sample read data were consolidated, and gene expression levels across all samples were determined. Gene expression matrices were analyzed using DESeq2 (v1.42.1). Gene expression differences were determined using |log2Fold Change| > 1 and *P*-value < 0.05 as criteria (26).

### 2.9 Western blot analysis

Colon specimens were disrupted in Radio Immunoprecipitation Assay buffer with protease inhibitors. Protein quantification was carried out via the bicinchoninic acid method. Equivalent protein aliquots underwent separation through sodium dodecyl sulfate polyacrylamide gel electrophoresis (SDS-PAGE) electrophoresis and were subsequently blotted onto polyvinylidene fluoride membranes employing a standard wet transfer protocol. Membranes were treated with 5% casein at ambient conditions for 60 minutes, followed by incubation with specific primary antibodies at 4 °C throughout the night (27). The primary antibodies used were against *Rorc*, *Foxp3*, *IL-17*, *IL-10*, and β-actin (as a loading control). Subsequently, the membranes underwent a 1-hour room-temperature incubation with HRP-linked secondary antibodies. Enhanced chemiluminescence substrate was employed to visualize protein bands. After quantification by ImageJ densitometry, the relative expression levels of the target proteins were normalized to β-actin. All experiments were independently repeated three times.

### 2.10 Statistical analysis

Statistical significance was determined through two-way ANOVA and multiple comparison analyses with GraphPad prism (v9.5.1), all data were presented as “mean ± standard error of mean (SEM)”, and *P* < 0.05 was considered statistically significant (*P < 0.05; **P < 0.01; ***P < 0.001; ****P < 0.0001). Statistical significance for two-group comparisons was determined by an unpaired two-tailed Student’s t-test, while one-way ANOVA (with Tukey’s or Dunnett’s test) or the Kruskal-Wallis H test (with Dunn’s test) was used for multi-group comparisons of parametric and non-parametric data, respectively. Shannon and Simpson indices were computed with the “diversity” function from the “vegan” R package (v2.6-10) and selected to estimate the changes of α-diversity of gut microbial communities with the t test. Based on the taxonomic composition of microbial communities at the species level, inter-group differences were calculated. The “anosim” function from the “vegan” package was used to compute *P*-values and *r*-values. Principal coordinates analysis (PCoA) was conducted via the “stats” package (v3.5.0) and visualized with “ggplot2” (v3.2.0). Heatmap was performed using the “ComplexHeatmap” package (v2.12.0). Mediation analysis was performed using the “mediation” package (v4.5.0) and “plyr” package (v1.8.9). Perform Kyoto Encyclopedia of Genes and Genomes (KEGG) and Gene Ontology (GO) enrichment analyses using the “clusterProfiler” (v4.10.1) and “org.Rn.eg.db” (v3.18.0) packages. Additionally, cytoscape (v3.9.1) was employed for graphically representing the intercorrelations among different bacterial species. Spearman’s correlation matrix was computed, with statistical significance set at *P* < 0.05.

## 3 Result

### 3.1 Network pharmacology analysis on the effect of TW on Th17 cell differentiation in RA

Using the method of network pharmacology, we predicted that TW has a total of 586 therapeutic targets and RA has 6,059 disease targets. After matching and mapping these targets, we found that there are 358 intersecting targets (**Figure 1A**). We used the STRING database and the CytoNCA tool of cytoscape to calculate the betweenness centrality (BC) values of the intersecting targets. The core targets were determined based on the BC values. The top five core targets are *SRC, PTGS2, IL6, TNF*, and *STAT3* (**Figure 1B**). The targets of TW in the treatment of RA are mainly enriched pathways in cancer, alzheimer disease, neuroactive ligand−receptor interaction, and Th17 cell differentiation pathways (**Figure 1C**). GO enrichment analysis revealed that the targets were primarily localized to the postsynapse, cell body, and receptor complexes. The primary biological processes encompassed include the cellular response to nitrogen compounds and hormonal stimuli, along with the regulation of the MAPK cascade. The targets mainly participated in molecular functions such as protein kinase activity, kinase activity, and phosphotransferase activity with an alcohol group as acceptor (**Figure 1D**). Ultimately, we established a potential mechanism network of therapeutic mechanisms for RA using its key constituents and associated targets (**Figure 1E**). The results revealed that 25 active ingredients were screened from TW, 18 of which were associated with 27 genes in the Th17 cell differentiation pathway.

**Figure 1:**
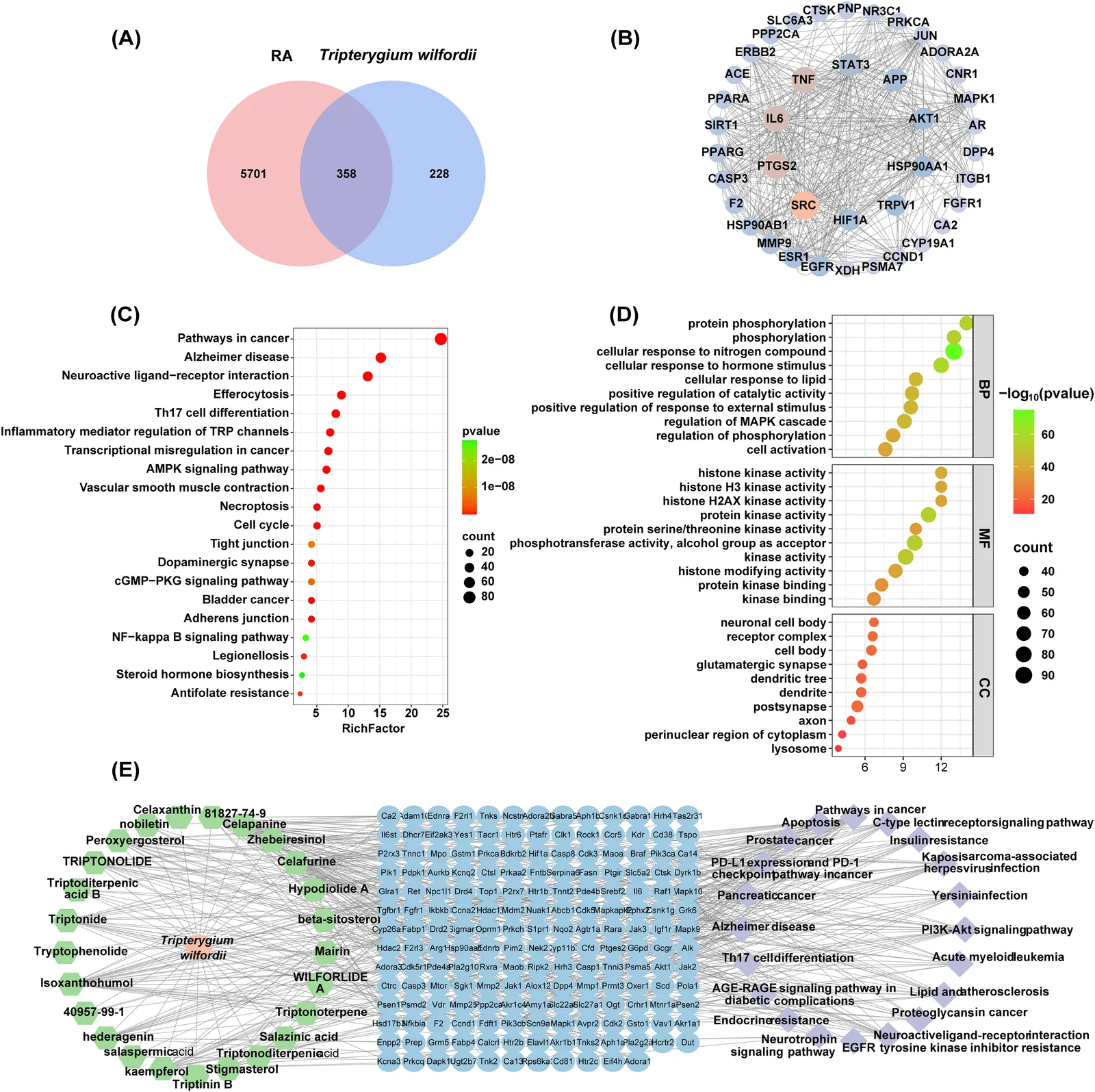
A network pharmacology-based investigation of the potential mechanisms of *Tripterygium wilfordii* in treating rheumatoid arthritis. **(A)** Venn-diagram showing 358 overlapping targets between rheumatoid arthritis (RA) and *Tripterygium wilfordii* (TW). **(B)** Hub targets of TW against RA, including SRC, PTGS2, IL6, TNF, and STAT3. **(C)** KEGG-pathway enrichment analysis of TW targets for RA treatment, revealing significant enrichment of the Th17-cell-differentiation pathway. **(D)** GO enrichment analysis of TW targets for RA intervention, showing enrichment for histone-kinase-activity molecular function. **(E)** Integrated network of TW-active components-target genes-disease pathways. Green nodes represent the bioactive compounds of TW, orange nodes represent the TW, blue nodes indicate target genes, and purple nodes denote disease-related pathways, respectively.

### 3.2 TW significantly ameliorated RA-associated symptoms in the RA rats

To evaluate the therapeutic effects of TW on RA, CFA rats were administered TW at concentrations of 5g/kg/d and 2.5 g/kg/d, and then the arthritis-related characteristics were examined. From one day before the intervention to day 28 of the intervention, body weight, arthritis score, paw length, and paw thickness were monitored every 2–3 days (**Figure 2A**). Compared with CFA rats, rats administered MTW-5g/kg/d exhibited significant increases in BW (one-way ANOVA, *P* < 0.05, **Figure 2B**), decreases in arthritis score (one-way ANOVA, *P* < 0.0001, **Figure 2C**), increases in paw length (one-way ANOVA, *P* < 0.01, **Figure 2D**), and decreases in paw thickness (one-way ANOVA, *P* < 0.001, **Figure 2E**). Serial photographic documentation of rat paws during the treatment period demonstrated significantly enhanced therapeutic effects with 5g/kg/d TW administration compared to the 2.5 g/kg/d dose (**Figure 2F**). Assessment of immune organ indices and inflammatory cytokines revealed that MTW treatment significantly suppressed spleen (*P* < 0.01) and thymus indices in CFA rats. It also reduced serum levels of IL-6, TNF-α, and IL-10, while conversely elevating IL-1β in the CFA+LTW group (one-way ANOVA, **Figures 2G, H**). The TW treatment mitigated CFA severity proportionally to dosage, validating its therapeutic effect in rat models. Western blot analysis (**Figure 2I**) revealed that TW treatment upregulated colonic RORC and FOXP3 expression (**Figures 2J, K**), while significantly decreasing the RORC/FOXP3 ratio (ANOVA, *P* < 0.01, **Figure 2L**), showing a trend consistent with the Con group. Furthermore, IL-10 expression was elevated in both CFA+MTW and CFA+LTW groups, with the higher levels observed in CFA+LTW (ANOVA, *P* < 0.01, **Figure 2M**). Conversely, IL-17 levels were reduced in the CFA+MTW group (**Figure 2N**).

**Figure 2:**
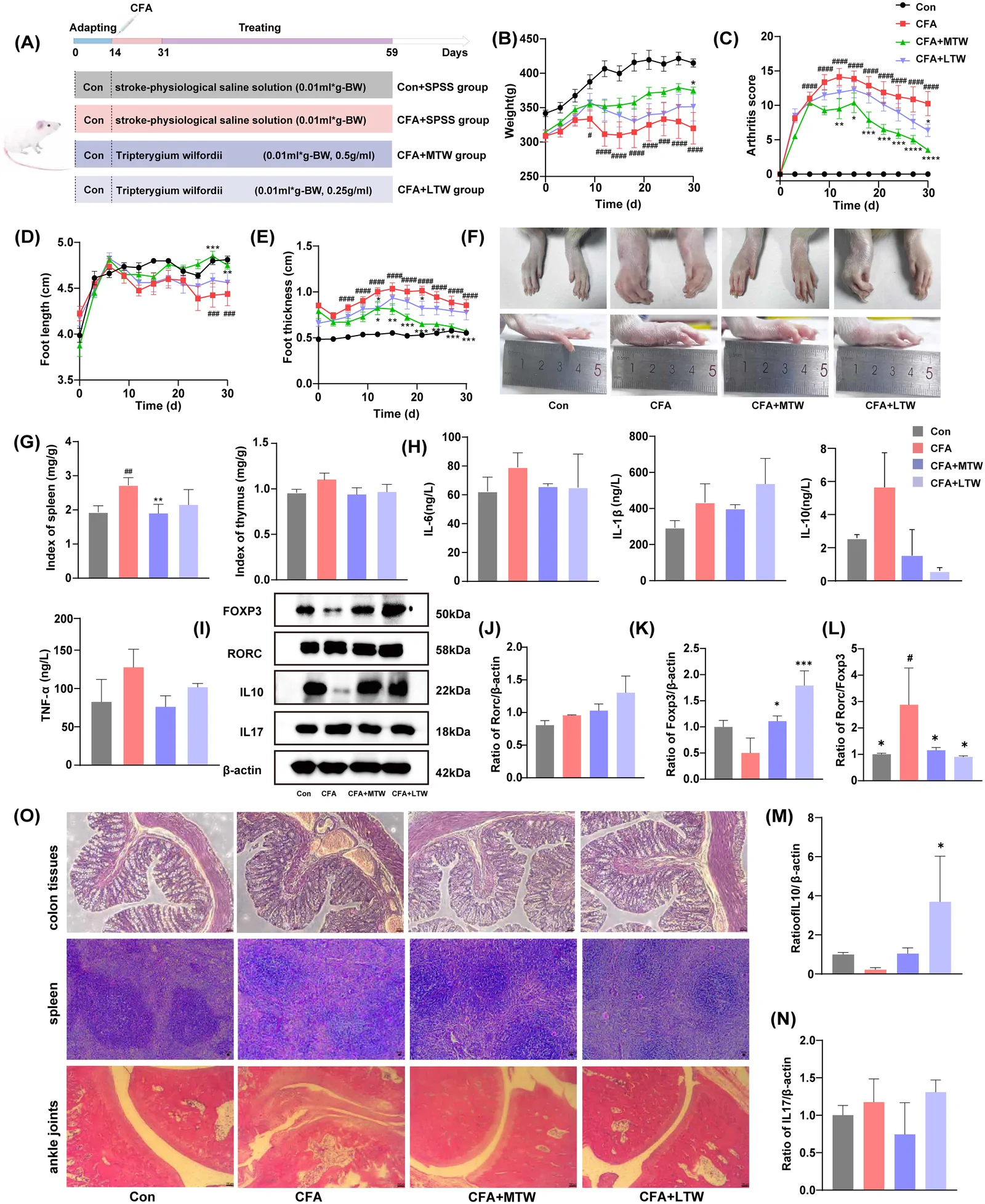
Oral administration of TW to RA rats promoted weight gain, alleviated paw swelling, reduced serum inflammation, and improved damage in the colon, spleen, and ankle joints. **(A)** Schematic diagram showing the experimental design for exploring the potential mechanisms of *Tripterygium wilfordii* (TW) in the treatment of CFA-induced rheumatoid arthritis (RA) in rats. Rat body weight **(B)**, arthritis score **(C)**, paw length **(D)**, and paw thickness **(E)** were measured every 2–3 days after primary immunization. At the end of the experiment, body weight, arthritis score, paw length and paw thickness were significantly improved in the MTW group compared with the CFA group, whereas no significant alterations were observed in the LTW group. **(F)** On day 17, rats were assigned to different treatment groups and received 28 consecutive days of drug administration. Representative photographs of rat paws from each group were captured after the final treatment, showing obvious alleviation of paw swelling in the MTW group. **(G)** At the end of the experiment, the spleen index was markedly decreased in the MTW group, while no significant difference was detected for the thymus index. **(H)** Serum levels of inflammatory cytokines including IL-6, IL-1β, TNF-α and IL-10 were reduced in the MTW group relative to the CFA group, without reaching statistical significance. Western blot analysis of FOXP3, RORC, IL-10 and IL-17 protein abundances in colon tissues **(I)**, together with corresponding quantitative analysis **(J–N)**. **(O)** Representative H&E-stained images of colon, spleen, and ankle-joint tissues. *p < 0.05, **p < 0.01, ***p < 0.001, ****p < 0.0001 compared with the CFA group; #p < 0.05, ##p < 0.01, ###p < 0.001, ####p < 0.0001 compared with the Con group.

### 3.3 TW significantly alleviated damage in the colon, spleen, and ankle joints of RA rats

Rats with RA exhibited edema of the colonic mucosa, degeneration of the crypts, abnormal structure of the splenic follicles, white pulp expanding outward with blurred boundaries from the red pulp, and obvious pathological changes in the ankle joints, including cartilage surface damage and narrowed joint space. After treatment with TW, the colonic mucosa of the treated rats was restored, with reduced inflammation. The spleen structure and the morphology of the splenic follicles became clear, with distinct boundaries from the red pulp. The structure of the ankle joint cartilage was improved, inflammatory cell infiltration was reduced, and the joint space was widened (**Figure 2O**).

### 3.4 TW can alleviate gut microbiota imbalance in RA rats

To investigate how TW influences gut microbial communities in RA rats, fecal metagenomic sequencing was conducted. The analysis revealed that TW decreased alpha diversity (Shannon and Simpson indices) at the phylum and species levels relative to the CFA group (**Figures 3A, B**), suggesting TW reduces gut microbial diversity in RA rat populations. PCoA was employed to visualize differences in community structure at both the phylum and species levels. Although gut microbiota composition showed no significant differences at the phylum level (**Figure 3C**), notable changes emerged at the species level (*P* < 0.01, **Figure 3D**). Additionally, the F/B ratio (Firmicutes/Bacteroidetes) is often used as an indicator of gut health. Therefore, we evaluated gut health status following TW treatment based on phylum-level. The CFA group (3.811% ± 0.807%) showed a 28.5% reduction in F/B ratio compared to Con group (5.329% ± 2.056%). The F/B ratio showed a 68.5% increase (6.421 ± 2.718) in the CFA+MTW group and a 93.4% increase (7.37 ± 2.559) in the CFA+LTW group relative to the CFA group. The CFA group exhibited an enrichment of *Firmicutes* and a depletion of *Bacteroidetes* relative to the Con group (**Figure 3E**). After TW treatment, the F/B ratio increased, suggesting that TW can improve gut health in RA rats. According to LDA (linear discriminant analysis), the gut microbiota in the TW-treated group exhibited a closer resemblance to that of the Con group (**Figure 3F**). Compared to the Con group, the CFA group showed a marked rise in *Lactobacillus johnsonii* (Con: 10.68% ± 3.017%, CFA: 14.25% ± 4.915%, CFA+MTW: 13.12% ± 4.745%, CFA+LTW: 12.66% ± 3.860%) and *GGB79683 SGB21436* (Con: 5.664% ± 2.895%, CFA: 12.45% ± 3.725%, CFA+MTW: 5.994% ± 1.587%, CFA+LTW: 13.75% ± 2.945%) but a decreased abundance of *Ligilactobacillus murinus* (Con: 18.52% ± 6.654%, CFA: 9.443% ± 3.470%, CFA+MTW: 16.98% ± 4.581%, CFA+LTW: 22.03% ± 7.809%) in the gut microbiota. However, after TW treatment, the CFA+MTW and CFA+LTW groups showed reduced abundances of *Lactobacillus johnsonii* and *GGB79683 SGB21436* and an elevated abundance of *Ligilactobacillus murinus*, reflecting a trend consistent with that of the Con group (**Figure 3G**).

**Figure 3:**
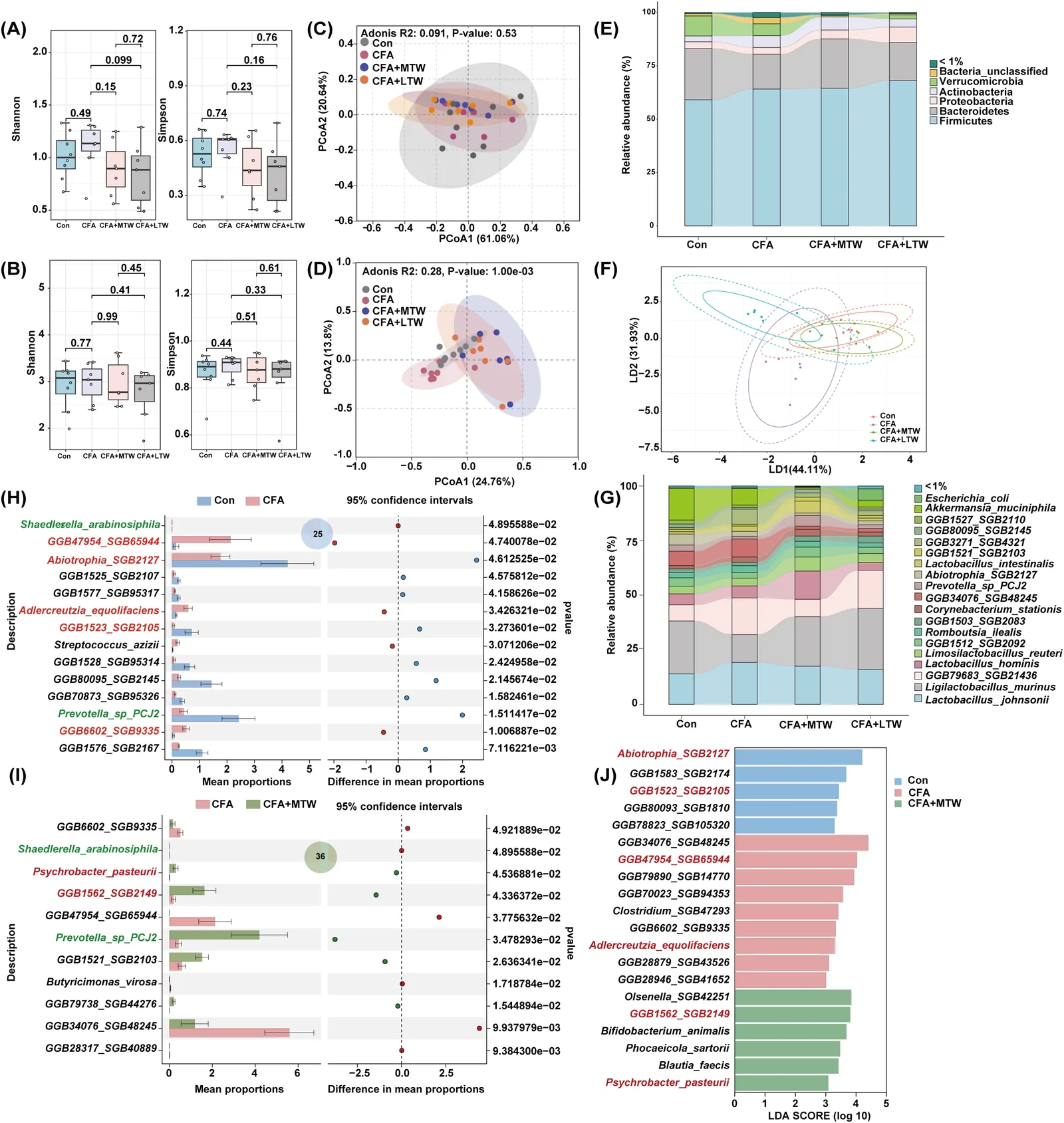
The gut microbiome showed significant alterations following TW administration in RA rats. **(A)** Gut microbiota diversity at the phylum level was assessed using Shannon and Simpson indices among Con, CFA, CFA+MTW, and CFA+LTW groups. **(B)** Gut microbiota diversity at the species level was assessed using Shannon and Simpson indices among Con, CFA, CFA+MTW, and CFA+LTW groups. **(C)** PCoA showing gut microbiota phyla taxonomy. **(D)** PCoA analysis of gut microbiota communities at species level. **(E)** Histogram of species composition at phylum level. **(F)** Linear discriminant analysis of species-level gut microbiome taxonomy. **(G)** Histogram of species composition at species level. **(H)** Species-level gut microbiome profiles were compared between the Con and CFA groups, with a 95% confidence interval. **(I)** Species-level gut microbiome profiles were compared between the CFA and CFA+MTW groups, with a 95% confidence interval. **(J)** LEfSe analysis based on species-level taxonomic classification. **p < 0.01, ***p < 0.001, ****p < 0.0001.

To identify potential microbial biomarkers associated with RA regulation, we performed statistical analysis of taxonomic and functional profiles (STAMP) and linear discriminant analysis effect size (LEfSe) at the species level (LDA > 3.7, *p* < 0.05). The STAMP analysis revealed that TW administration altered particular gut microbial abundances. A marked elevation of *Prevotella* sp. *PCJ2* occurred in the CFA+MTW group (3.672% ± 1.226%) versus the CFA group (0.9458% ± 0.535%); meanwhile, *Schaedlerella arabinosiphila* exhibited a distinct pattern (Con: 0.0002% ± 0.0002%; CFA: 0.0027% ± 0.0011%; CFA+MTW: 0.00043% ± 0.0004%; CFA+LTW: 0.00037% ± 0.0003%, **Figures 3H, I**). LEfSe analysis showed that the dominant species in the CFA+MTW group were *Olsenella SGB42251* (1.367% ± 1.160%), *GGB1562 SGB2149* (1.402% ± 0.508%), *Bifidobacterium animalis* (0.954% ± 0.729%), *Phocaeicola sartorii* (0.153% ± 0.082%), *Blautia faecis* (0.511% ± 0.229%), and *Psychrobacter pasteurii* (0.253% ± 0.101%, **Figure 3J**). Among these, *Psychrobacter pasteurii* and *GGB1562 SGB2149* may be key species associated with TW’s therapeutic effects on RA.

### 3.5 TW can significantly enhance amino acid metabolites in RA rats

To investigate TW’s influence on intestinal metabolites in RA rats, untargeted metabolomics sequencing was performed on fecal from each group. Using PCoA analysis, we observed a clear separation in intestinal metabolites between TW-treated and untreated RA rats (*P* < 0.001, **Figure 4A**). Between the Con and CFA groups, 106 differential metabolites (DMs, VIP > 1, *P* < 0.05; Mann-Whitney test) were identified, with 50 showing upregulation and 56 downregulation (**Figure 4B**). Compared to the CFA group, the CFA+MTW group exhibited 201 differential metabolites were detected, with 140 upregulated and 61 downregulated (**Figure 4C**). Sixty-one overlapping metabolites were detected across the Con vs CFA and CFA vs CFA+MTW comparisons. Analysis of the top 40 metabolites revealed distinct distribution patterns (**Figure 4D**). In both the Control and CFA+MTW groups, there was a significant increase in Enkephalinamide-Leu, 5-Nonyltetrahydro-2-oxo-3-furancarboxylic acid, Vasicinone, 6-(4-Sulfophenyl)nonanoic acid, Labetalol, and Glutamylleucine levels when compared to the CFA group. In contrast, Pyrithiobac, Lyciumoside VIII, and Goshonoside F3 showed higher levels in the CFA group versus both Con and CFA+MTW groups (**Figure 4E**). Furthermore, our analysis revealed significant enrichment of DMs in the tryptophan metabolism and neuroactive ligand-receptor interaction pathways between the Con and CFA groups (**Figure 4F**). In contrast, DMs identified between the CFA and CFA+MTW groups showed prominent enrichment in steroid hormone biosynthesis, arachidonic acid metabolism, neuroactive ligand-receptor interactions, aldosterone synthesis and secretion, and bile secretion pathways (**Figure 4G**). Compared to the CFA group, the levels of tryptophol (Con: 31973.27, CFA: 7205.50, CFA+MTW: 22338.95, CFA+LTW: 34156.11), tryptamine (Con: 2.66×10^6^, CFA: 4.19×10^5^, CFA+MTW: 1.86×10^6^, CFA+LTW: 3.69×10^6^), and 5-hydroxykynurenamine (Con: 8898.91, CFA: 0, CFA+MTW: 2608.34, CFA+LTW: 16148.51) were increased in both the CFA+MTW and CFA+LTW groups, whereas 4-(2-aminophenyl)-2,4-dioxobutanoic acid (Con: 5.33×10^6^, CFA: 1.07×10^7^, CFA+MTW: 8.71×10^6^, CFA+LTW: 8.59×10^6^) was markedly decreased (*P* < 0.01, ANOVA, **Figure 4H**). Among metabolites enriched in the neuroactive ligand-receptor interaction pathway, the concentrations of Ile-Ile-Ile-Pro (Con: 1.09×10^5^, CFA: 4.70×10^3^, CFA+MTW: 3.58×10^4^, CFA+LTW: 2.12×10^5^) and LysoPA(P-16:0/0:0) (Con: 3.48×10^6^, CFA: 2.00×10^6^, CFA+MTW: 6.15×10^6^, CFA+LTW: 3.42×10^6^) were significantly increased following TW treatment (*P* < 0.01). Conversely, 1-(2-amino-3-hydroxyphenyl)ethanone sulfate, pregnenolone, and PA(8:0/20:4(5Z,8Z,11Z,14Z)-OH (20)) exhibited marked reductions (**Figure 4I**).

**Figure 4:**
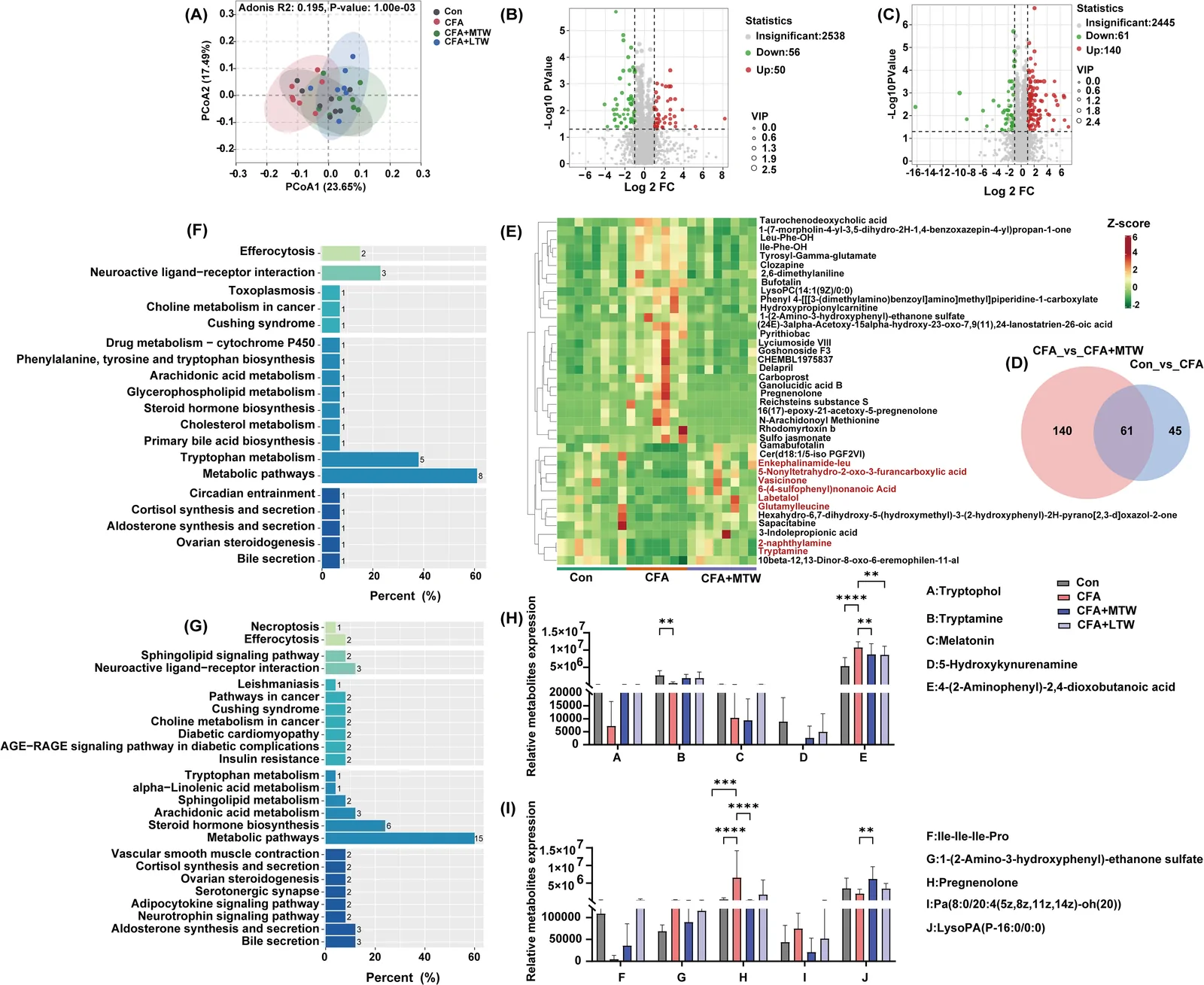
Identification of key differential gut metabolites and enriched pathways reveals the therapeutic mechanisms of TW in RA treatment. **(A)** PCoA analysis discriminates gut metabolite profiles among different groups. **(B)** Volcanic distribution map for differences in DMs among the Con and CFA groups. **(C)** Volcanic distribution map for differences in DMs among the CFA and CFA+MTW groups. **(D)** Venn diagram showing differential gut metabolites between the Con vs CFA and CFA vs CFA+MTW comparisons. **(E)** DMs were identified between Con and CFA groups, and between CFA and CFA+MTW groups, with the top 40 metabolites displayed in this heatmap. KEGG pathway enrichment analyses for the Con versus CFA comparison **(F)** and the CFA versus CFA+MTW comparison **(G)** both revealed significant enrichment in the glycerophospholipid and tryptophan metabolism pathways. **(H, I)** Key differential gut metabolites enriched in tryptophan metabolic pathways and neuroactive ligand-receptor interaction pathways.

### 3.6 TW significantly enhances tryptophan metabolism in serum of RA rats

TW may influence RA progression by modulating gut microbiota and altering serum metabolite levels. Therefore, we conducted untargeted metabolomic sequencing on serum samples from each group of rats. A total of 135 serum differential metabolites were identified between the Con and CFA groups, among which 72 were up-regulated and 63 were down-regulated (**Figure 5A**). In the comparison between the CFA and CFA+MTW groups, 302 serum differential metabolites were detected, with 118 metabolites being up-regulated and 184 down-regulated (**Figure 5B**). Principal component analysis (PCA) revealed a clear separation in serum metabolites between the CFA group and the other groups. Notably, the CFA+MTW group exhibited a metabolic profile closer to that of the Con group (**Figure 5C**). This separation was supported by R2Y and Q2 intercepts both above 0.70, indicating a robust model with strong predictive capability (**Figure 5D**). Following TW administration, 105 DMs exhibited concordant directional changes with the Con group, comprising 46 downregulated and 59 upregulated metabolites. Among the upregulated metabolites, 17 were identified as amino acids and their derivatives, including N-acetyl-L-tyrosine, L-4-chlorotryptophan, formyllysine, and N-formyl-L-methionine. The top 40 DMs ranked by variable importance in projection (VIP) scores revealed significant TW-induced upregulation of aminomethyl fluorescein, Phenylalanylhistidine, Amidinophenylpyruvic acid, Alanylasparagine, N-formyl-L-methionine, and aminofructose 6-phosphate. Notably, serum concentrations of aspartyl-isoleucine (Con: 1.81×10^5^, CFA: 5.23×10^5^, CFA+MTW: 2.15×10^5^, CFA+LTW: 2.98×10^5^), Asp-leu (Con: 3.98×10^5^, CFA: 1.20×10^6^, CFA+MTW: 5.85×10^5^, CFA+LTW: 7.18×10^5^), virginiamycin m1(Con: 3.03×10^5^, CFA: 1.51×10^6^, CFA+MTW: 6.65×10^5^, CFA+LTW: 1.23×10^6^), Asp-ile-arg (Con: 6.84×10^4^, CFA: 4.48×10^5^, CFA+MTW: 1.78×10^5^, CFA+LTW: 2.73×10^5^), and Endomorphin-1(Con: 7.39×10^4^, CFA: 3.86×10^5^, CFA+MTW: 1.85×10^5^, CFA+LTW: 2.66×105) were markedly elevated in RA rats but normalized to Con group levels after TW intervention (**Figure 5E**).

**Figure 5:**
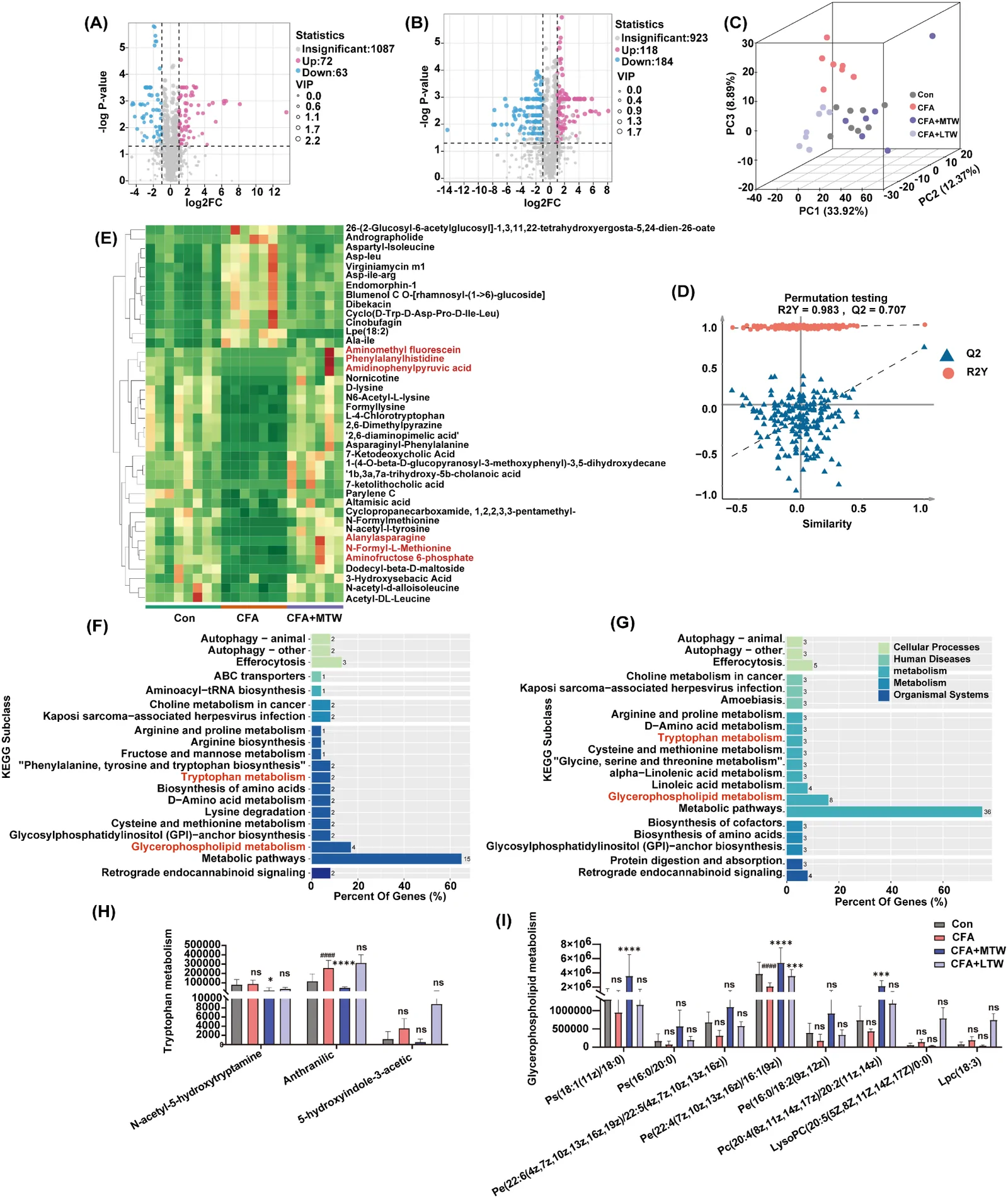
Identification of key serum differential metabolites and enriched pathways revealed the therapeutic mechanisms of TW in RA treatment. **(A)** Volcanic map of DMs between the Con and CFA groups. **(B)** Volcanic plot displaying DMs for CFA and CFA+MTW groups. **(C)** PCA analysis discriminates serum metabolite profiles among different groups. **(D)** Permutation test analysis of OPLS-DA. **(E)** DMs were identified between Con and CFA groups, and between CFA and CFA+MTW groups, with the top 40 metabolites displayed in this heatmap. KEGG enrichment analysis of serum differential metabolites for the Con versus CFA **(F)** and CFA versus CFA+MTW **(G)** comparisons revealed significant enrichment in tryptophan metabolism and glycerophospholipid metabolism pathways. **(H-I)** Key differential serum metabolites enriched in tryptophan metabolic pathways and Glycerophospholipid metabolism pathways. ***p < 0.001, ****p < 0.0001 compared with the CFA group; ###p < 0.001, ####p < 0.0001 compared with the Con group.

Subsequently, we conducted enrichment analysis of differential metabolites among groups to identify the pathways modulated by TW treatment. Differential metabolites common to both the Con vs CFA and CFA vs CFA+MTW comparisons were primarily enriched in glycerophospholipid and tryptophan metabolism (**Figures 5F, G**). Based on the above analyses, we quantified key amino acid levels to evaluate the impact of TW treatment on serum metabolites in RA rats. The findings suggest that TW mitigates RA by modulating key amino acid and lipid metabolic pathways. Building upon the aforementioned analyses, we quantitatively assessed three differentially regulated metabolites enriched in tryptophan metabolism and eight lipid-class metabolites evaluate TW’s impact on the serum metabolome in RA rats. Our results demonstrated that TW treatment significantly decreased serum levels of N-acetyl-5-hydroxytryptamine, anthranilate, and 5-hydroxyindole-3-acetic acid, while increasing concentrations of lipid-class metabolites (**Figures 5H, I**). These findings collectively indicate that TW exerts therapeutic effects against RA through coordinated modulation of both tryptophan metabolism and lipid metabolic pathways.

### 3.7 TW regulates IL-17 signaling pathway in synovial tissue of CFA-induced RA rats

To clarify the processes mediating TW’s protective impact on RA synovial tissue, RNA sequencing was conducted to examine synovial gene expression patterns. Transcriptomic profiling revealed distinct gene expression patterns across experimental groups. Compared to Con group, the CFA group exhibited 385 DEGs, consisting of 197 downregulated and 188 upregulated genes, with the top five most significant DEGs being *Kcnj3*, *Parp4*, *LOC100364958*, *LOC100911962*, and *Dsg2*. In the CFA+MTW group relative to CFA, we identified 454 DEGs (212 downregulated, 242 upregulated), featuring *Prss1*, *LOC691532*, *Parp4*, *Rag1*, and *Csn1s1* as the most prominent changes. Similarly, the CFA+LTW group showed 463 DEGs compared to CFA (150 downregulated, 313 upregulated), with *LOC120100609*, *Prss3b*, *Cpb1*, *Gp2*, and *Myh4* demonstrating the most significant alterations (**Figure 6A**). These findings suggest treatment-specific modulation of gene expression profiles of synovial tissue in the CFA-induced RA model.

**Figure 6:**
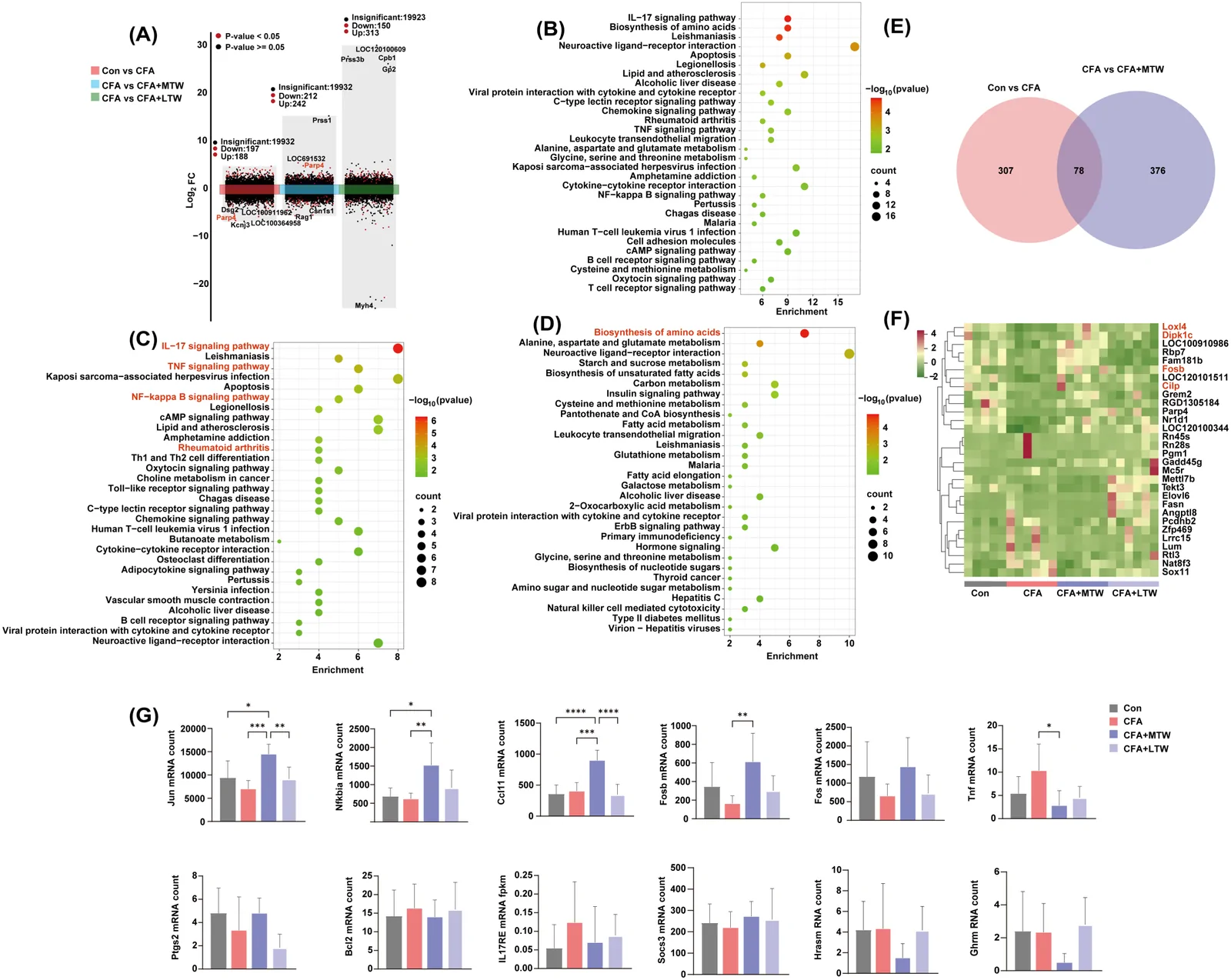
Synovial transcriptome analysis revealed differential gene expression in the synovium of RA rats following TW treatment. **(A)** Volcano plot of DEGs in Con and CFA, CFA and CFA+MTW, CFA+LTW groups. **(B)** KEGG pathway enrichment was conducted on DEGs from CFA and CFA+MTW groups. **(C)** KEGG functional enrichment analysis of differentially upregulated genes in CFA+MTW compared to CFA. **(D)** KEGG functional enrichment analysis of differentially downregulated genes between CFA and CFA+MTW groups. **(E)** Venn diagram displaying common DEGs between Con and CFA groups, and CFA and CFA+MTW groups. **(F)** Heatmap displaying the top 40 common DEGs between Con and CFA groups, and between CFA and CFA+MTW groups. **(G)** Genes showing altered expression demonstrated significant enrichment in IL-17-NF-κB and JAK-STAT signaling pathways. *p < 0.05, **p < 0.01, ***p < 0.001, ****p < 0.0001.

KEGG pathway analysis identified distinct signaling patterns among the different experimental groups. In the Con versus CFA comparison, DEGs exhibited significant enrichment in the neuroactive ligand-receptor interaction pathway (**Supplementary Figure S1A**). Upregulated genes showed prominent enrichment in ribosome biogenesis in eukaryotes (**Supplementary Figure S1B**), while downregulated genes were predominantly associated with neuroactive ligand-receptor interactions (**Supplementary Figure S1C**). DEGs predominantly gathered in key biological activities, including the regulation of epithelial cell proliferation and response to corticosteroid, localized mainly within the receptor complex, and exhibited molecular functions including signaling receptor activator activity and receptor ligand activity (**Supplementary Figure S1D**).

For the CFA versus CFA+MTW comparison, significant pathway enrichments included the biosynthesis of amino acids, IL-17 signaling pathway, and leishmaniasis (**Figure 6B**). The CFA+MTW group showcased a notable enhancement in the IL-17 signaling pathway, as well as in various rheumatoid arthritis-related pathways, including TNF signaling, NF-κB signaling, and the core RA pathway (**Figure 6C**). Conversely, the most significantly downregulated pathway was biosynthesis of amino acids (**Figure 6D**). To investigate the key DEGs predominantly affected by TW treatment, we performed comparative analysis of DEGs between Con vs CFA and CFA vs CFA+MTW groups, identifying 78 overlapping DEGs (**Figure 6E**). Among the top 40 DEGs displayed, *Loxl4*, *Dipk1c*, *Fosb*, and *Cilp* exhibited markedly low expression in the CFA group but were significantly elevated in both Con and CFA+MTW groups (**Figure 6F**). Differential analysis revealing increased expression of *Jun*, *Nfkbia*, *Ccl11*, and *Fosb* in the CFA+MTW group compared to the CFA group linked to the IL-17 signaling pathway (*P* < 0.01, ANOVA, **Figure 6G**), while *Tnf* mRNA expression was notably downregulated (*P* < 0.05, ANOVA). While *Fos* and *Ptgs2* were upregulated in the CFA+MTW group, their expression levels mirrored those in the Con group. In contrast, *Bcl2* and *IL17RE* showed significant downregulation following treatment. These findings suggest that TW treatment may modulate IL-17-associated inflammatory responses by restoring the expression of key regulatory genes toward normal levels.

### 3.8 Integrated multi-omics analysis reveals the potential mechanisms of TW in alleviating RA

#### 3.8.1 The combined metabolomic and transcriptomic study shows the links between DMs and DEGs in RA

Integrated network pharmacology, metabolomics, and transcriptomics analyses identified several key pathways, including the biosynthesis of amino acids, TNF signaling pathway, IL-17 signaling pathway, NF-κB signaling pathway, and rheumatoid arthritis. Genes enriched in these pathways were subjected to Spearman correlation analysis with differentially expressed metabolites, revealing 28 metabolites exhibiting strong correlations (|*r*| > 0.6, *p* < 0.01). Notably, *Jun*, *Nfkbia*, *Ptgs2*, and *TNF* were identified as shared RA-related targets in both network pharmacology and differentially expressed gene analyses. The findings revealed that *Jun* and *Nfkbia* displayed a marked positive association with several compounds, including LysoPA(P−16:0/0:0), GPGro(4:0/18:1), Tetroxoprim, Tryptamine, 5−[1−(cyclobutanecarbonyl)piperidine−4−carbonyl]−1,5−diazacycloundecan−2−one, M−Coumaric acid, PA(18:3 (10,12,15)−OH(9)/i−12:0), and Sitosterol 3−O−(6’−O−stearyl−beta−D−glucoside) (all *P* < 0.05, |*r|* > 0.6), but a significant negative correlation with PE−NMe(18:1(9Z)/20:4(5Z,8Z,11Z,14Z)), PE−NMe(20:4(8Z,11Z,14Z,17Z)/18:0), 1−(benzenesulfonyl)−3−(2−phenoxyethyl)pyrrolidine, and N−Myristoyl Arginine. *Ptgs2* exhibited a positive association with LysoPA(P−16:0/0:0) and Ganoderic acid V, while showing a negative correlation with 1−(benzenesulfonyl)−3−(2−phenoxyethyl)pyrrolidine (all *p* < 0.05, |*r|* > 0.6). Additionally, *Tnf* displayed a positive correlation with PE−NMe(18:1(9Z)/20:4(5Z,8Z,11Z,14Z)), Acetyl−Ser−Asp−Lys−Pro, N−Myristoyl Arginine, Betulinic acid and a negative correlation with Phytosphingosine, Dg(18:0/20:4(8z,11z,14z,17z)−2oh(5s,6r)/0:0), Dg(18:0/20:5(7z,9z,11e,13e,17z)−3oh(5,6,15)/0:0), PA(18:3(10,12,15)−OH(9)/i−12:0), Tyrosylhydroxyproline (all *P* < 0.05, |*r|* > 0.6, **Figure 7A**).

**Figure 7:**
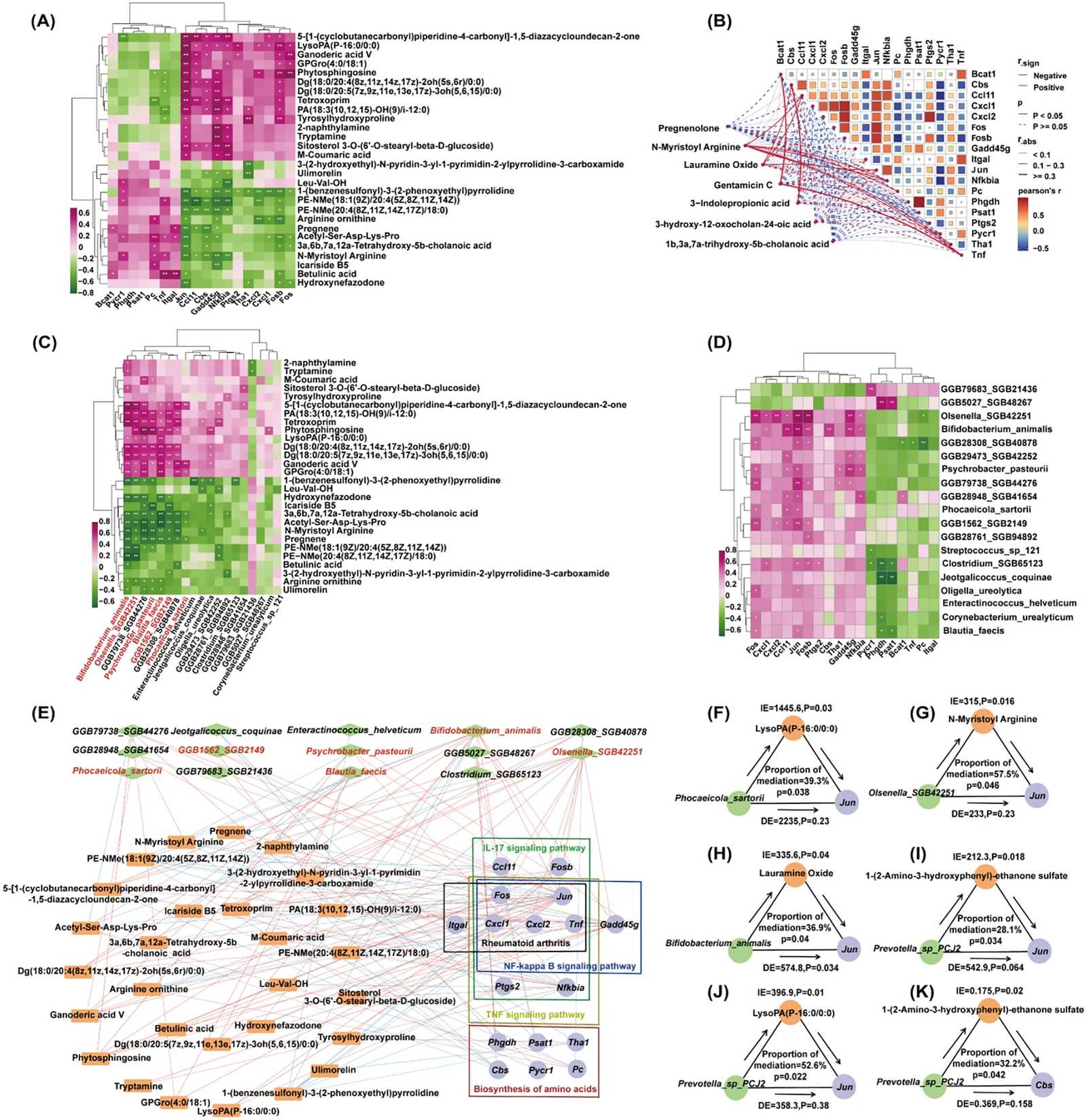
Multi-omics integration unravels the mechanisms underlying TW therapy in RA. **(A)** Spearman correlation heatmap between key genes and differential metabolites. **(B)** Associations between the six shared metabolites (in feces and serum) and key genes. **(C)** Heatmap illustrating relationships between GM and DMs. **(D)** Heatmap of associations between GM and key genes. **(E)** Network showing the influence of key microbes on the rheumatoid arthritis, IL-17 signaling pathway, NF-κB signaling pathway, TNF signaling pathway, and amino-acid biosynthesis. green nodes indicate GMs, orange nodes denote DMs, and purple nodes correspond to DEGs. Pathway affiliations are distinguished by colored borders surrounding each node. Red lines representing positive associations (*r* > 0.5) and blue lines indicating negative correlations (*r* < -0.5), where all displayed connections meet statistical significance thresholds (*P* < 0.05). **(F)** *Phocaeicola_sartorii* upregulates *Jun* expression by enhancing LysoPA(P-16:0/0:0) production (39.3% mediation, *P* = 0.038). **(G)** *Olsenella SGB42251* promotes *Jun* expression through increased biosynthesis of N-myristoyl arginine (57.5% mediation, *P* = 0.046). **(H)** *Bifidobacterium animalis* significantly elevates *Jun* expression via Lauramine Oxide generation (36.9% mediation, *P* = 0.04). *Prevotella* sp *PCJ2* contributes to *Jun* expression by elevating **(I)** 1-(2-amino-3-hydroxyphenyl)-ethanone sulfate (28.1%, Pmediation = 0.034) and **(J)** LysoPA (P-16:0/0:0) (52.6%, *p* = 0.022). Additionally, *Prevotella* sp *PCJ2* enhances *Cbs* expression by promoting 1-(2-amino-3-hydroxyphenyl)-ethanone sulfate production (32.2% mediation, *p* = 0.042) **(K)**. IE (Indirect Effect) represents the causal pathway through the mediator. DE (Direct Effect) represents the unmediated regulatory impact of microbial species on gene expression.

Small-molecule metabolites (e.g., short-chain fatty acids, amino acids, bile acids, and lipids) are absorbed by intestinal epithelial cells, enter the portal venous circulation, and ultimately reach the liver, joint synovial tissue and systemic circulation. Seven differentially abundant metabolites were consistently detected in both gut and serum samples, including Pregnenolone, N−Myristoyl Arginine, Lauramine Oxide, Gentamicin C, 3−Indolepropionic acid, 3−hydroxy−12−oxocholan−24−oic acid, 1b,3a,7a−trihydroxy−5b−cholanoic acid. Among them, Gentamicin C demonstrated a strong positive correlation with *Bcat1* and *Tnf*. Conversely, 3-Indolepropionic acid exhibited a strong positive correlation with *Cbs*, a key enzyme in the amino acid biosynthesis pathway. All correlations were statistically significant (*P* < 0.05, |*r*| > 0.5, **Figure 7B**).

#### 3.8.2 Integrated metagenomic and metabolomic analyses revealed associations between gut microbiota and DMs in RA rats

A Spearman correlation analysis between key GM and DMs uncovered six metabolites that showed significant associations with the dominant species within the CFA+MTW treatment group, particularly *Bifidobacterium animalis*, *Olsenella SGB42251*, *Psychrobacter pasteurii*, *Blautia faecis*, *GGB1562 SGB2149*, and *Phocaeicola sartorii*. The data revealed significant associations between these dominant microbial species and specific metabolites. Notably, positive correlations were observed with oxidized lipid including PA(18:3(10,12,15)-OH(9)/i-12:0), tetroxoprim, DG(18:0/20:4(8Z,11Z,14Z,17Z)-2oh(5S,6R)/0:0), and DG(18:0/20:5(7Z,9Z,11E,13E,17Z)-3oh(5,6,15)/0:0), along with ganoderic acid V (all *P* < 0.05, |*r*| > 0.6). Conversely, the six key GM were negatively correlated with several metabolites, including the bile acid derivative 3a,6b,7a,12a−tetrahydroxy−5b−cholanoic acid; peptide metabolites such as acetyl-Ser-Asp-Lys-Pro and N-myristoyl arginine; and steroid compounds pregnene (all *P* < 0.05, |*r*| > 0.6, **Figure 7C**).

#### 3.8.3 Integrated microbiome and transcriptome profiling reveals host-microbe interactions in RA rats

To elucidate the gut microbiota-synovium axis in RA pathogenesis, we conducted Spearman correlation analysis between gut microbial taxa and synovial gene expression profiles. Focusing on genes enriched in key inflammatory pathways, we identified significant microbiota-gene associations. The genus *Olsenella SGB42251* demonstrated strong positive correlations with multiple *Fos*, *Cxcl1*, *Cxcl2*, *Ccl11*, *Jun*, *Fosb*, *Gadd45g*, *Nfkbia*, while showing negative association with Pc. *Bifidobacterium animalis* exhibited consistent positive correlations with chemokine genes (*Cxcl1*, *Ccl11*) and stress response factors (*Jun*, *Fosb*, *Gadd45g*, *Nfkbia*), along with the transsulfuration enzyme *Cbs*. Notably, *Psychrobacter pasteurii* and *Phocaeicola sartorii* shared positive associations with the AP-1 transcription factor components (*Fos*, *Jun*) and eosinophil chemokine *Ccl11*, with *P. pasteurii* additionally correlating with *Tha1* and *Nfkbia*. The unclassified Firmicutes species *GGB1562 SGB2149* showed broad inflammatory gene associations (*Fos*, *Cxcl2*, *Ccl11*, *Jun*, *Fosb*). In contrast, *Blautia faecis* displayed dual regulation - positive correlations with *Fos* and *Jun* but negative associations with serine biosynthesis enzymes (*Phgdh*, *Psat1*, all *P* < 0.05, |*r*| > 0.6, **Figure 7D**).

#### 3.8.4 Multi-omics profiling unveils the anti-rheumatoid arthritis mechanisms of TW

Integrated network analysis combining metagenomic (14 GMs), metabolomic (28 DMs), and transcriptomic (17 DEGs) data identified crucial microbe-host interactions. Fourteen microbial species showed significant correlations with both metabolic alterations and gene expression changes (Spearman’s correlation, *P* < 0.05), suggesting their potential involvement in systemic regulation. Of particular interest, six dominant species in the CFA+MTW group include *Psychrobacter pasteurii*, *Phocaeicola sartorii*, *GGB1562 SGB2149*, *Olsenella SGB42251*, *Bifidobacterium animalis*, and *Blautia faecis* - demonstrated robust interactions with host genes and were functionally associated with five key metabolic pathways: the rheumatoid arthritis, IL-17 signaling pathway, amino acid biosynthesis, TNF signaling pathway, and NF-κB signaling pathway (*P* < 0.05, |*r*| > 0.6, **Figure 7E**). Mediation analysis identified several significant microbial metabolite-mediated pathways. Specifically, LysoPA(P-16:0/0:0) served as a positive mediator between *Phocaeicola_sartorii* and upregulated *Jun* expression (39.3% mediation, Pmediation = 0.038; **Figure 7F**). Similarly, *Olsenella SGB42251* enhanced *Jun* expression through N-Myristoyl Arginine mediation (57.5% mediation, Pmediation = 0.046; **Figure 7G**). Lauramine Oxide functioned as a positive mediator linking *Bifidobacterium animalis* to *Jun* upregulation (36.9% mediation, Pmediation = 0.04; **Figure 7H**), while *Bifidobacterium animalis* also exhibited a significant direct effect on *Jun* expression (DE = 574.8, *P* = 0.034), indicating its capacity to directly promote *Jun* expression. *Prevotella* sp *PCJ2* contributes to *Jun* expression by elevating 1-(2-amino-3-hydroxyphenyl)-ethanone sulfate (28.1%, Pmediation = 0.034) and LysoPA(P-16:0/0:0) (52.6%, Pmediation = 0.022, **Figures 7I, J**). Furthermore, *Prevotella* sp *PCJ2* also promoted *Cbs* expression through 1-(2-amino-3-hydroxyphenyl)-ethanone sulfate mediation (32.2% mediation, Pmediation = 0.042; **Figure 7K**).

## 4 Discussion

Based on network pharmacology predictions of TW’s potential mechanisms against RA, experimental validation in a CFA-induced rat model demonstrated marked anti-arthritic effects. The therapeutic efficacy was confirmed by the observed reductions in systemic inflammation scores, paw swelling, and immune organ indices, along with increased body weight and modulated serum inflammatory cytokine levels. Employed an integrated multi-omics approach to explore the interplay between gut microbial communities, gut metabolites, and synovial gene expression governance.

An imbalance in gut microbiota has been linked to the development of rheumatoid arthritis in previous studies (28, 29), to further investigate this connection, we conducted metagenomic sequencing on stool specimens from all test groups to examine how the composition of intestinal microbes differed between rats with induced RA and those receiving TW therapy. At both the phylum and species levels, we observed increased alpha diversity in the GM of CFA-induced RA rats. Notably, TW treatment significantly reduced these microbial diversity metrics. Furthermore, PCoA analysis revealed that the CFA+MTW group exhibited microbial community structures most similar to the Con group. These results demonstrate that TW effectively remodels gut microbiota composition toward a healthier state. Our results demonstrated significantly reduced abundance of *Prevotella* sp *PCJ2* and *Ligilactobacillus murinus* in the GM of RA rats, while higher abundance was observed in both Con and CFA+MTW groups. Previous studies have identified *Prevotella* as a key microbial genus influencing branched-chain amino acid (BCAA) metabolism, as it possesses enzymes involved in BCAA degradation, enabling utilization of these amino acids as energy sources (30). Notably, healthy individuals typically exhibit higher gut abundance of *Prevotella* species (31), which aligns consistently with our current findings. Tryptophan-derived metabolites indole-3-lactic acid (ILA) and indole-3-aldehyde (IAld) from *L. murinus* exhibit significant regulatory effects on intestinal health (32). These microbial tryptophan catabolites function as crucial intercellular signaling molecules (33). Current evidence indicates that ILA modulates immune responses during intestinal inflammation and enhances gut barrier homeostasis. Furthermore, 3-IAld administration has demonstrated protective efficacy against immune checkpoint inhibitor-induced colitis in murine models (34–36). In our study, *Bifidobacterium animalis*, *Phocaeicola sartorii*, *Blautia faecis*, and *Psychrobacter pasteuri*i were identified as the dominant gut microbial species following TW treatment. Previous research has demonstrated that *B. animalis* ameliorates RA by modifying gut microbiota composition at phylum and genus levels and downregulating key histidine metabolites including 1-methyl-L-histidine and urocanate (37). These results suggest that TW may further ameliorate the progression of RA through these dominant microbial species. Methotrexate (MTX) is the first-line conventional synthetic DMARD for the clinical treatment of RA. Following low-dose MTX administration, the relative abundance of intestinal *Firmicutes* increases while that of Bacteroidetes decreases; the opposite trend is observed in the high-dose MTX group. The relative abundance of *Firmicutes* is positively correlated with fecal DAMPA excretion (38). Consistent with this finding, our study also detected an elevated ratio of Firmicutes to Bacteroidetes in RA rats receiving low-dose TW. Clinical cohort data further indicate that characteristic bacterial species including *Acetanaerobacterium elongatum*, *Cristiansella massiliensis* and *Gracilibacter thermotolerans* are significantly enriched in healthy subjects, whereas *Blautia gnavus* exhibits higher abundance in patients with early rheumatoid arthritis (39). Mechanistically, MTX exerts anti-inflammatory effects via multiple pathways: it inhibits *de novo* purine and pyrimidine synthesis and transmethylation reactions, blocks the nuclear translocation of NF-κB, and suppresses signal transduction through the JAK/STAT axis (40). By contrast, our experimental data demonstrate that TW alleviates RA progression by regulating tryptophan metabolism and inhibiting the IL-17-NF-κB signaling pathway within synovial tissues.

Although substantial evidence implicates the gut microbiota in the progression of RA (41–43), the underlying cellular and molecular mechanisms, along with the associated host pathways, remain elusive. Emerging research indicates the significant impact of gut microbial byproducts on immune system regulation and inflammation linked to immune responses (44–46). Butyrate, a microbial byproduct, inhibits autoimmune arthritis advancement by facilitating follicular regulatory T cell maturation in collagen-induced arthritis models (47, 48). Pathway analysis based on gut metabolite enrichment revealed that tryptophan metabolism, neuroactive ligand-receptor interactions, steroid hormone biosynthesis, and arachidonic acid metabolism were identified as key therapeutic pathways associated with TW treatment in the CFA-induced RA rat model. Metabolomic analysis revealed significant enrichment of tryptophan metabolism pathway metabolites, particularly tryptophol, tryptamine, and 5-hydroxykynurenamine, following treatment. Importantly, microbial metabolism of tryptophan yields tryptophol as a key bioactive metabolite, previous studies have established that microbial metabolism converts tryptophan to tryptophol, a bioactive metabolite demonstrating potent IFN-γ suppression (49). Tryptamine is primarily synthesized by gut microbiota (e.g., *Lactobacillus* and *Bifidobacterium* spp.) via the tryptophan decarboxylation pathway. Through its action on 5-HT4 receptors in proximal colonic epithelium, tryptamine enhances anion-dependent fluid secretion, thereby modulating gastrointestinal transit. This mechanism represents a crucial small molecule-mediated host-microbiota interaction (12, 50). Our findings identify pregnenolone, Ile-Ile-Ile-Pro, and 1-(2-amino-3-hydroxyphenyl)-ethanone sulfate as critical mediators of neuroactive ligand-receptor interactions in RA. Pregnenolone is synthesized from cholesterol via the catalytic activity of *CYP11A1* and can be further metabolized to cortisol, which exerts anti-inflammatory effects, or to dehydroepiandrosterone, which modulates immune responses (51). Immunological markers associated with macrophage polarization and inflammatory cytokine secretion demonstrate significant correlation with arachidonic acid metabolism (52).

Non-targeted metabolomics identified that TW’s anti-arthritic action is linked to modulating glycerophospholipid and tryptophan metabolic pathways. After TW treatment, metabolites enriched in the glycerophospholipid pathway (e.g., Ps(18:1(11z)/18:0), Pe(22:4(7z,10z,13z,16z)/16:1(9z))) were significantly increased, previous studies have demonstrated that glycerophospholipid metabolism participates in rheumatoid arthritis development through its regulatory effects on the IL-6/JAK/STAT inflammatory cascade (53). Gut-derived metabolites can enter the bloodstream via the portal venous system, intestinal lymphatic drainage, or epithelial-specific transporters. Metabolomic analysis revealed seven differential metabolites that were simultaneously detected in both serum and feces: pregnenolone, N-myristoyl arginine, lauramine oxide, gentamicin C, 3-indolepropionic acid, 3-hydroxy-12-oxocholan-24-oic acid, and 1β,3α,7α-trihydroxy-5β-cholanoic acid. Our data suggest that *Olsenella SGB42251* enhances *Jun*-mediated inflammatory signaling in synovial fibroblasts via upregulation of N-Myristoyl Arginine. *B. animalis* upregulates *Jun* gene expression in synovial tissue through modulation of lauramine oxide levels. N-Myristoyl Arginine is a modified amino acid formed through an amide bond between myristic acid (14-carbon saturated fatty acid) and arginine. Myristoylation is a key regulator of inflammatory responses dependent on the TRIF-related adaptor molecule (TRAM) and mediated by TLR4 (54). Lipopolysaccharide (LPS), a key glycolipid in Gram-negative bacterial membranes, triggers TLR4-mediated inflammatory signaling (55). Rowe noted that myristoylation influences TRAM-mediated TLR4 signaling regulation (56). TRAM localizes to the plasma membrane by interacting with TLR4 through its N-terminal myristoyl group. Upon recognition of dimerized TLR4, TRAM dissociates from TLR4 and the membrane, subsequently binds to TRIF, and facilitates the activation of the NF-κB and IRF3 signaling pathways. This signaling pathway plays a critical role in the activation of IRF3 and NF-κB, while also functioning as a negative feedback mechanism for TLR4 signaling to prevent the overproduction of inflammatory cytokines.

RA is propelled by a network of pro-inflammatory signaling cascades, in which the IL-17 and NF-κB pathways critically interact (57). Our transcriptomic analysis identified significant enrichment of DEGs in the IL-17 signaling pathway, NF-κB signaling pathway, and TNF signaling pathway. These pathway-related genes were activated following TW treatment, which is also consistent with previous research findings (58). Investigation of these phenomena through assessment of Suppressor of Cytokine Signaling 3 (SOCS3) expression in synovial tissues revealed marked overexpression in the treatment group. Research indicates that SOCS3, influenced by IL-17/NF-κB, serves as a negative regulator, suppressing JAK-STAT3 signaling pathways. SOCS3 binds to phosphorylated JAK1/JAK2, blocking STAT3 activation and subsequent pro-inflammatory gene expression (59, 60). Thus, while transcriptional upregulation of IL-17/NF-κB pathway genes may reflect residual pathway activity, the concomitant SOCS3 overexpression likely uncouples transcription from functional output, resulting in net anti-inflammatory effects. Our findings demonstrate that *Ghr* and *Hras* (Con: 4.2 ± 1.241, CFA: 4.333 ± 1.783, CFA+MTW: 1.5 ± 0.563, CFA+LTW: 4.167 ± 0.946), genes enriched in the JAK-STAT signaling pathway, exhibited lower expression levels in the CFA+MTW treatment group, further validating this conclusion. Overall, our findings demonstrate that TW supplementation significantly ameliorated key inflammatory parameters in RA rats through gut-joint axis, including the levels of pro- and anti-inflammatory cytokines (IL-6, IL-1β, TNF-α, and IL-10) and systemic inflammation scores. Beyond these physiological benefits, the results revealed a marked remodeling of the gut microbiota, characterized by the specific suppression of detrimental bacterial species and a concurrent enrichment of beneficial strains. Importantly, we observed concomitant alterations in the gut metabolome, serum metabolome, and synovial gene expression profiles (**Figure 8**), which were linked to the observed mitigation of inflammation.

**Figure 8:**
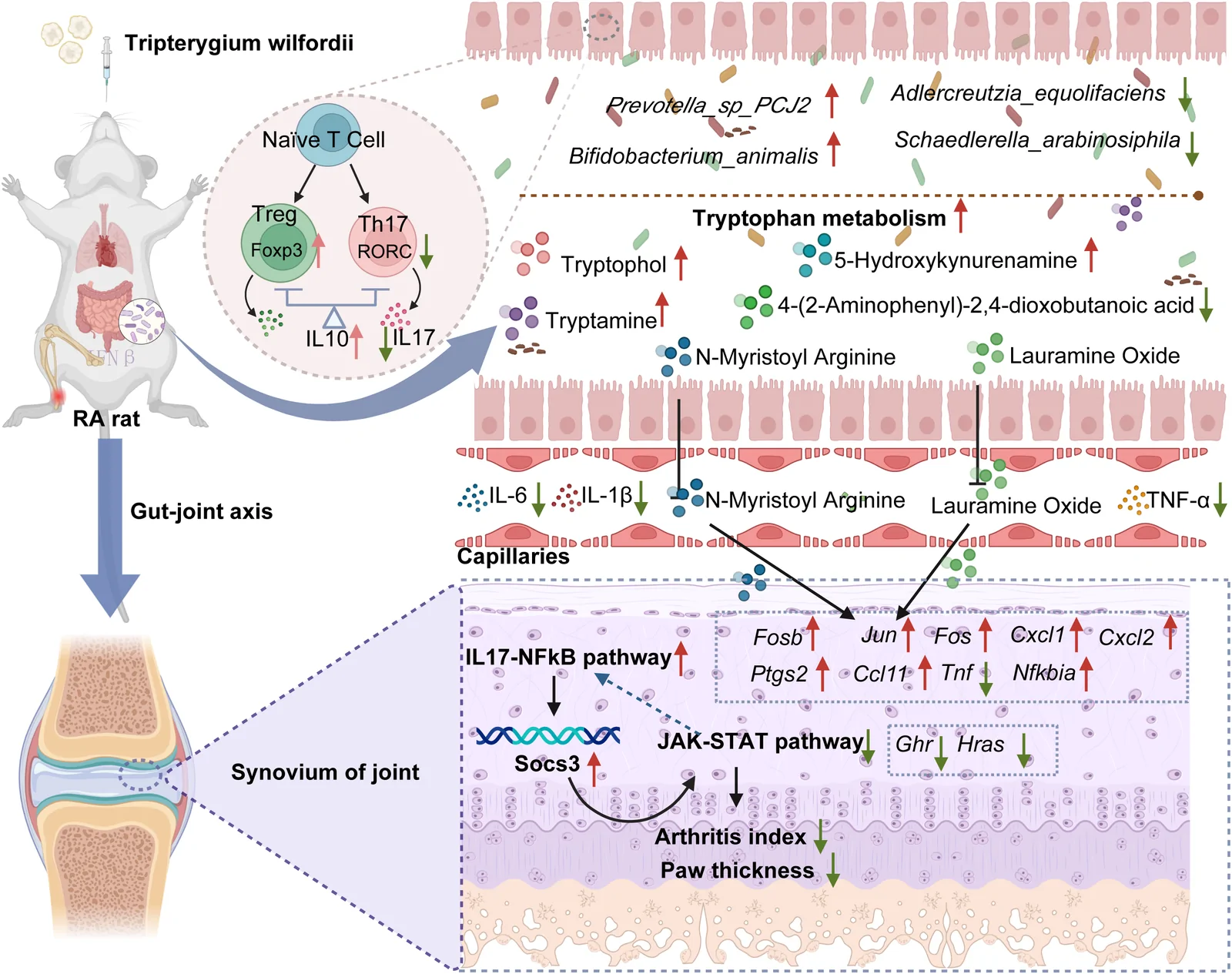
The underlying mechanism by which TW mediates the treatment of rheumatoid arthritis via the gut-joint axis, which involves in reprogramming gut microbiota, restoring tryptophan metabolism, and remodeling synovial gene expression.

Several limitations of the present study should be acknowledged. Although the integration of metagenomics, metabolomics, and transcriptomics provided insights into the potential gut–joint regulatory axis mediated by TW, these multi-omics findings primarily represent associative relationships and do not establish direct biological causality. Mediation analysis supported the plausibility of the proposed sequential gut–metabolite–joint signaling cascade; however, it cannot completely exclude potential confounding factors. Therefore, the identified microbial taxa, metabolites, and regulatory pathways should be considered mechanistic hypotheses requiring further validation. In future studies, we will adopt strategies including in-vivo animal experiments with key candidate strains and in-vitro cellular intervention assays to verify whether candidate bacteria can exert independent anti-rheumatoid-arthritis effects, and to clarify the causal relationships between candidate microbes and joint inflammatory signaling. In addition, although synovial transcriptomic analysis revealed enrichment of IL-17–NF-κB and JAK-STAT signaling pathways and alterations in related genes after TW treatment, these findings provide evidence of pathway-associated changes rather than direct confirmation of pathway activation or inhibition. Future studies using fecal microbiota transplantation, targeted metabolite intervention, pathway-specific inhibitors or agonists, and genetic manipulation approaches are needed to clarify the causal roles of candidate microbes, metabolites, and inflammatory pathways in TW-mediated protection against RA. Finally, findings derived from rodent models should be interpreted cautiously and cannot be directly extrapolated to human RA. Validation of key microbial and metabolic signatures in independent clinical cohorts will be necessary to further evaluate the translational potential of TW targeting the gut–joint axis.

## 5 Conclusion

In conclusion, our findings demonstrate that TW ameliorates RA in rats via regulation of the gut-joint axis and the mechanism involved in reprogramming gut microbiota, restoring tryptophan metabolism, and remodeling synovial gene expression. Specifically, TW enriches beneficial bacteria (e.g., *Prevotella* sp *PCJ2*, *Bifidobacterium animalis*) while reducing *Adlercreutzia equolifaciens* and *Schaedlerella arabinosiphila*, thereby improving tryptophan metabolism elevating Tryptophol and Tryptamine, lowering 4-(2-Aminophenyl)-2,4-dioxobutanoic acid. Gut-derived metabolites including N-myristoyl arginine and Lauramid oxide were positively correlated with the transcriptional levels of inflammatory genes such as *Jun* in synovial tissues. These metabolic signatures may participate in the regulation of the IL-17-NF-κB inflammatory cascade. Meanwhile, upregulated Socs3 in synovium could inhibit JAK-STAT signaling via negative feedback, thereby reducing the secretion of pro-inflammatory cytokines including IL-6, IL-1β and TNF-α and alleviating joint inflammatory injury.

## Data Availability

Metagenomic sequencing datasets for fecal samples and transcriptome data for joint synovial tissues in our study have been deposited into NCBI’s Sequence Read Archive (SRA) database with the BioProject number: PRJNA1362644. Fecal metabolomic datasets were stored in the figshare database: DOI 10.6084/m9.figshare.28838525.
