# Supplementary material for: Tripterygium wilfordii ameliorates complete freund adjuvant-induced rheumatoid arthritis through modulating the gut-joint axis
Source: Front Immunol. 2026 Oct 8;17:1828110. doi: 10.3389/fimmu.2026.1828110 (PMC13686877; doi:10.3389/fimmu.2026.1828110)
Supplement: Supplementary file 1 [file DataSheet1.docx]

**Supplementary Material**

***Tripterygium wilfordii* Ameliorates Complete Freund Adjuvant-Induced Rheumatoid Arthritis through Modulating the Gut-Joint Axis**

Caili Zhang^1, 2^, Qingru Chen^1, 2^, Zheng Sun^1, 2^, Tiantian Wu^1, 2^, Yujie Zhang^1, 2^, Binbin Huang^3^, Shenghai Huang^4, *^, Yan Zhang^5, *^, Maozhen Han^1, 2, *^

^1^ School of Life Sciences, Anhui Medical University, Hefei, Anhui, 230032, China.

^2^ Microbial medicinal resources development research team, Anhui Provincial Institute of Translational Medicine, Hefei, Anhui, 230032, China.

^3^ School of Public Health, Anhui Medical University, Hefei, Anhui 230032, China.

^4^ Department of Microbiology, The Key Laboratory of Microbiology and Parasitology of Anhui Province, The Key Laboratory of Zoonoses of High Institutions in Anhui, School of Basic Medical Sciences, Anhui Medical University, Hefei, Anhui 230032, China.

^5^ School of Life Sciences, Hefei Normal University, Hefei, Anhui 230601, China.

^*^ Corresponding author E-mail: hanmz@ahmu.edu.cn; zhangyanwind@163.com; huangshh68@aliyun.com

**Figure S1.** **Transcriptomic analysis of the effects of synovial gene expression in normal controls and CFA-induced RA rats.** **(A)** KEGG pathway enrichment analysis of DEGs. **(B)** KEGG pathway enrichment analysis of down-regulated genes. **(C)** KEGG pathway enrichment analysis of up-regulated gene. **(D)** GO pathway enrichment analysis of DEGs.
